# Electroacupuncture exerts antipruritic and anti-inflammatory effects on atopic dermatitis by activating CB2 receptor

**DOI:** 10.1186/s13020-025-01102-4

**Published:** 2025-05-26

**Authors:** Wenqiang Ge, Xin Liu, Xuefei Hu, Yang Yang, Hongxiang Chen, Ouyang Zhan-Mu, Shiying Lin, Yanzhen Li, Peiling Li, Qing Tian, Xianghong Jing, Man Li

**Affiliations:** 1 School of Basic Medical Science, Key Laboratory of Neurological Diseases of Hubei Province and National Education Ministry, Tongji Medical College, Huazhong University of Science and Technology, 13 Hangkong Road, Wuhan, 430030 China; 2 Department of Dermatology Union Hospital, Tongji Medical College, Huazhong University of Science and Technology, Wuhan, China; 3 Institute of Acupuncture and Moxibustion China Academy of Chinese Medical Sciences (CACMS), Beijing, China

**Keywords:** Electroacupuncture, CB2 receptor, Atopic dermatitis, Chronic itch, Inflammation

## Abstract

**Background:**

The therapeutic benefits of electroacupuncture (EA) for atopic dermatitis (AD) are recognized, yet the underlying mechanisms remain elusive. Given the side effects associated with clinical CB2 receptor (CB2R) agonists used in AD treatment, our study seeks to elucidate EA's role in modulating CB2R in lesional skin and its impact on antipruritic and anti-inflammatory responses using an AD mouse model.

**Methods:**

The AD model was induced with MC903, and EA was applied to'Qu chi'(LI11) and'He gu'(LI4) acupoints, corresponding to the neck dermatome. Mice were assessed for scratching behavior and scoring atopic dermatitis score every other day. Immunohistochemistry and immunofluorescence evaluated epidermal thickness, inflammatory cell infiltration, and CB2R expression. Meanwhile, RT-qPCR detected the expression of inflammatory factors, their receptors, and cannabinoid metabolizing enzymes. The study used both wild-type and CB2R knockout (CB2R^−/−^) mice to clarify CB2R's role in EA's treatment of AD.

**Results:**

EA treatment effectively mitigated chronic itching and AD-like symptoms, especially the proliferation of mast cells and CD4^+^ T cells. Additionally, EA treatment was found to reduce the expression of IL4, IL13, and IL31 in the skin lesions, as well as the expression of their receptors IL4R and IL31R in the dorsal root ganglia of the neck, contributing to its anti-inflammatory action. Moreover, EA augmented the expression of CB2R and regulated endocannabinoid metabolic enzymes. Furthermore, using CB2R^−/−^ mice, it was found that the antipruritic and anti-inflammatory effects of EA were impaired. EA inhibited ERK phosphorylation in lesional skin, which was also reversed in CB2R^−/−^ mice.

**Conclusion:**

EA exerts therapeutic effects on persistent itch and skin inflammation in AD mice by activating CB2R, thereby inhibiting mast cell and CD4^+^ T cell proliferation and the expression of associated inflammatory factors, as well as downstream ERK phosphorylation.

**Supplementary Information:**

The online version contains supplementary material available at 10.1186/s13020-025-01102-4.

## Introduction

Chronic itch persisting for more than 6 weeks, as a prominent symptom of chronic skin inflammatory diseases like atopic dermatitis (AD), severely disrupts patients' daily activities and profoundly affects their quality of life [1, 2]. AD is a prevalent chronic inflammatory condition of the skin, predominantly driven by Th2 cells [3, 4]. While numerous treatment options exist, some come with side effects, there are limited combination therapies that can address both inflammation and itching simultaneously. Electroacupuncture (EA), a significant component of traditional Chinese medicine, has gained traction as a complementary treatment [5, 6]. Previous experiments and clinical research have demonstrated that EA can effectively relieve the clinical manifestations of individuals suffering from AD [7], but the precise mechanisms through which EA confers its therapeutic effects are yet to be comprehensively understood and clarified.

The endocannabinoid system comprises a complex network, including two primary cannabinoid receptors (CB1R and CB2R), as well as key ligands such as anandamide (AEA) and 2-arachidonoylglycerol (2-AG) [8]. The system is further regulated by metabolic enzymes [9], including N-acyl phosphatidylethanolamine-phospholipase D (NAPE-PLD) and diacylglycerol lipase β (DAGL β), which act as endogenous cannabinoid synthetases, and fatty acid amide hydrolase (FAAH) and monoacylglycerol lipase (MAGL), which serve as endogenous cannabinoid hydrolases [10]. These components orchestrate the intricate balance of endocannabinoid synthesis and degradation, underscoring their pivotal role in a spectrum of physiological and pathological processes of atopic dermatitis [11].CB2R stands out as a pivotal element within this system, exerting a significant influence on inflammatory processes as evidenced by recent studies [12, 13]. Clinical trials have substantiated the therapeutic potential of CB2R agonists in treating AD [14]. Animal studies further demonstrate that CB2R agonists can markedly reduce inflammation associated with AD, while the application of CB2R antagonists tend to exacerbate the condition [15]. The therapeutic benefits of CB2R are mediated through mechanisms involving the afferent pathway [16, 17] and local immune cell regulation via the extracellular signal-regulated kinase (ERK) pathway [11]. EA exerts an antinociceptive effect by activating CB2R at the site of inflammation [18]. As a result, we hypothesize that EA may modulate immune cell activity and cytokine release through CB2R, resulting in anti-inflammatory and antipruritic effects.

It is widely recognized that AD patients'chronic pruritus can cause scratching behavior, which in turn can exacerbate dermatitis and further intensify pruritus [19]. Within the context of AD, type 2 cytokines such as interleukin- 4 (IL4), IL13, and IL31 are linked to the escalation of skin inflammation [20, 21]. Additionally, mice with elevated levels of IL4 or IL13 in their skin manifest symptoms reminiscent of AD and experience significant persistently itching sensations [22]. IL31, recently identified as a cytokine that induces itching, has been associated with the characteristic itchiness of AD [23]. Single-cell RNA sequencing (scRNA-seq) has indicated that sensory neurons in mouse express the IL4Rα subunit, a component common to receptors for IL4 and IL13, which is co-expressed in various sensory neurons implicated in pruritus [24]. Research has demonstrated that IL31R antagonist can markedly alleviate itching in individuals with AD [25]. Mast cell (MC) degranulation has been considered as a pivotal trigger for itchiness in allergic conditions, playing a central role in the inflammatory response [26]. Recent studies have revealed that IL13 and IL31, previously believed to be generated by TH2 cells, can also be secreted by mast cells [27]. This discovery suggests that mast cells may have a more extensive role in the development of chronic itching than previously thought. The activation of CD4^+^ T cells lead to the secretion of substantial amounts of Th2 cytokines, which have pro-inflammatory effects and can further aggravate the symptoms of AD [28, 29]. Despite these advances in understanding the immunological aspects of AD, the relationship between EA and its potential effects on these immunological targets is not yet fully established.

In our investigation, we employed the mouse model of AD to explore the potential of EA treatment in reducing skin inflammation and itch. Subsequently, we focused on the impact of EA on critical cellular and molecular components of AD, including mast cells, CD4^+^ T cells, and cytokines IL4, IL13, and IL31, along with their respective receptors. Notably, we investigated whether EA could enhance the expression of CB2R and modulate the endocannabinoid metabolic enzymes within AD skin lesions. By employing CB2R knockout (CB2R^−/−^) mice, we elucidated the role of CB2R in the antipruritic and anti-inflammatory effects mediated by EA. Furthermore, we explored the hypothesis that EA might attenuate the activation of the ERK pathway via CB2R engagement. Our comprehensive approach aimed to reveal the underlying mechanisms through which EA confers its therapeutic benefits in AD, emphasizing the pivotal role of the peripheral CB2R-ERK signaling in the therapeutic efficacy of EA treatment.

## Materials and methods

### Animals

Adult male C57BL/6 J mice were obtained from Beijing Vital River Laboratory Animal Technology and male CB2 receptor knockout (CB2R^−/−^) mice on C57BL/6 background aged 6–8 weeks from Jackson Laboratories (Strain #:005786). Mice were housed in separate cages under a 12-h light/dark treatment, the animals had unrestricted access to food and water. The experimental protocols were rigorously reviewed and endorsed by the Animal Care and Use Committee at Huazhong University of Science and Technology.

### Chronic itch mouse model induced by MC- 903 for atopic dermatitis

Three days before the start of the experiment, the neck skin of the mice was depilated, as reported in previous studies. Subsequently, to establish an AD model, MC903 (2 nmol/100 μl in ethanol, Sigma) or vehicle (ethanol) was topically applied to the neck skin of mice once a day for 7 consecutive days. After the modeling process concluded (on day 8), the lesional skin and the corresponding cervical dorsal root ganglion were excised for subsequent analyses.

### EA treatment

A total of 48 male C57BL/6 J mice were randomly assigned to four distinct groups(control, AD, AD + EA, AD + Sham EA) to evaluate EA’ s efficacy, while a total of 36 male CB2R^−/−^ mice and 36 wild-type littermates were divided into six subgroups (WT control, WT AD, WT AD + EA; CB2R^−/−^ control, CB2R^−/−^ AD, CB2R^−/−^ AD + EA) to specifically assess CB2R’ s role. EA treatment was applied to the left'Quchi'(LI11) and'Hegu'(LI4) acupoints, commencing on the second day post-induction of the model and continuing every other day up to the eighth day, totaling four sessions. EA was administered for 30 min at an intensity of 3 mA and a frequency of 100 Hz, using a modified Han’ s Acupoint Nerve Stimulator (LH202) with a constant current output [6]. The selection of LI11 and LI4 was based on their established efficacy in alleviating pruritus in mice.

The LI11 and LI4 acupoints were inserted with needles to a depth of 2–3 mm, aligning with the depth ratios customary in human acupuncture standards [18]. The LI11 acupoint is identified in the concavity at the lateral edge of the elbow crease upon complete elbow flexion. While LI4 is positioned at the temporal midpoint along the second metacarpal on the back of the hand [5]. Throughout the EA process, the mice were positioned in a clear plastic laminating bag without restrain, allowing them remained motionless and displayed no evident signs of distress. In the AD + sham EA group, needles were positioned at the LI11 and LI4 acupoints on the same side without electrical activation or manual needle adjustment. EA treatment was performed in the morning, followed by placing the mice in the itch behavior assessment apparatus to facilitate environmental adaptation.

### Scratching behavior

To capture scratching behavior, each mouse was individually placed in a distinct Plexiglas observation chamber with a transparent lid for a three-day adaptation period, with each period lasting 30 min [30]. Behavioral recordings were captured using a digital camcorder. During the itch behavior assessments conducted every other day, mice were positioned in the testing chamber for an initial 30 min acclimatization period, followed by filming their scratching behavior for 60 min. Throughout the recording process, the experimenter exited the laboratory room to maintain a quiet environment. The recorded videos were reviewed, and the number of scratching bouts was independently counted by two blinded experimenters who were unaware of the experimental groups. A scratching bout was defined as a series of consecutive scratching actions by the hindlimb aimed at the lesional skin area, terminating either upon the mouse engaging in toe-biting or licking, or when the hindlimb was repositioned on the floor [31].

### Scoring atopic dermatitis (SCORAD) score

The clinical assessment was conducted utilizing an adapted version of Scoring Atopic Dermatitis (SCORAD) instrument [32]. The evaluation focused on four key dermatological parameters: erythema, oozing/crusts, edema/papulation, and excoriations. Each parameter was visually scored on a scale from 0 to 3 (0, none; 1, mild; 2, moderate; 3, severe) to measure the severity. Lesion scoring was executed once the mice were back in their cages, following the completion of the video recording of scratching behavior. This evaluation was carried out by two observers who were both trained and unaware of the treatment conditions.

### Histological evaluation

Mice were subjected to anesthesia using sodium pentobarbital (250 mg/kg, i.p.). Subsequently, the lesional skin was immediately excised under cold conditions. Samples were preserved in 4% paraformaldehyde (PFA) solution, encapsulated in paraffin and sectioned into 5-μm-thick slices using a microtome. A portion of these sections was stained with hematoxylin and eosin (H&E) for the assessment of tissue morphology, while toluidine blue staining was employed for mast cell identification. The measurements of epidermal thickness, as well as the counts of total mast cells and degranulated mast cells, were conducted within five high-power fields (× 20 magnification) per mouse [33]. Quantification of the stained cells was conducted using ImageJ software.

### Immunofluorescence labeling

For immunofluorescence, the lesional skin tissue samples were processed as detailed in Sect. 2.6. Antigen retrieval was executed through heating the samples in citrate buffer solution. After rinsing with 0.1 M PBS, sections were blocked with a solution containing 5% donkey serum and 0.2% Tween- 20 in PBS for one hour. Subsequently, they were subjected to incubation with primary antibodies including anti-Mouse CD4 (Proteintech, China) and anti-rabbit CB2R (Abcam, Cambridge, UK) at 4 °C overnight. Then, the sections underwent three rounds of washing with PBS for 5 min, followed by incubation with corresponding secondary antibodies obtained from Jackson ImmunoResearch: donkey anti-mouse IgG conjugated with Dylight 488 or donkey anti-rabbit IgG conjugated with Dylight 594. Finally, the nuclei staining was visualized with DAPI and images were captured using a Nikon confocal microscope.

### Western blotting

Protein lysates were derived from injured skin and corresponding cervical segment DRG of deeply anesthetized mice in each group. These lysates underwent separation via a 10% gradient sodium dodecyl sulfate–polyacrylamide gel electrophoresis (SDS-PAGE) and then transferred onto a polyvinylidene difluoride (PVDF) membrane (Bio-Rad). After a 2-h blocking period at 37 °C using non-fat dry milk, the membranes were incubated with primary antibodies at 4 °C overnight. The primary antibodies used in this research are enumerated as follows: CB2R(1:500, Abclonal Biotechnology), extracellular signal‒regulated kinase (ERK, 1:1000, Cell Signaling Technology) and phosphorylated extracellular signal‒regulated kinase (p-ERK, 1:1000, Cell Signaling Technology). Subsequently, secondary antibodies conjugated with horseradish peroxidase (1:10,000, Proteintech) were applied and incubated at 37 °C for a duration of 1 h. The quantification of the relative gray intensity was performed utilizing ImageJ software, thereby assessing the proteins’ relative expression levels.

### Real-time quantitative PCR

Dorsal root ganglia tissues (cervical level) and skin tissue were promptly collected from mice that had been deeply anesthetized. RNA extraction was isolated using Trizol reagent, and its concentration was measured with a Thermo Fisher Scientific spectrophotometer. Subsequently, RNA was transcribed into complementary DNA (cDNA) utilizing the Vazyme Biotech cDNA Reverse Transcription Kit. For RT-PCR, SYBR Green Master Mix (Vazyme Biotech) was used in combination with the cDNA and specific primers. The sequences of all PCR primers used in this study are detailed in Supplementary Table S1.

### Statistical analysis

All data were presented as mean ± standard error of the mean (S.E.M). Statistical analysis was performed utilizing Prism 9 software. Behavioral and biochemical indicators of wild-type mice were analyzed using one-way ANOVA and Tukey's multiple comparisons test. Two-way ANOVA and Sidak's multiple comparisons test were applied to analyze scratching behavior and biochemical indicators in both wild-type mice and CB2R^−/−^ mice. Statistical significance was determined at the threshold of *p* < 0.05.

## Results

### EA alleviates chronic itch and AD-like symptoms

**Fig. 1:**
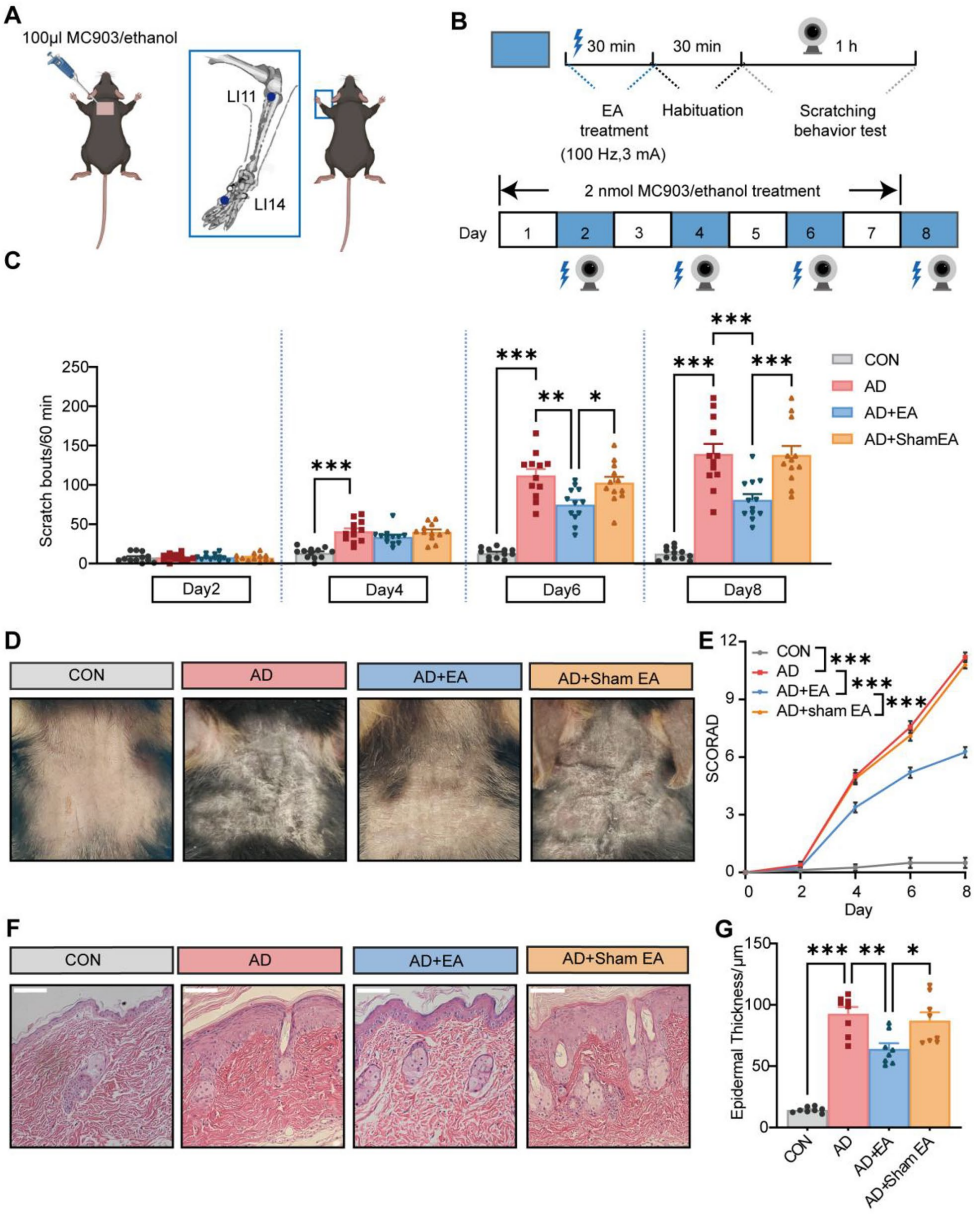
The impact of electroacupuncture (EA) on chronic itch and skin lesions of atopic dermatitis (AD). **A** Schematic illustration depicting the application of MC903 to the neck region (left) and the identification of acupoints Quchi (LI11) and Hegu (LI4) on the forelimb for EA treatment (right). **B** Experimental protocol outlining the induction of the AD model, the timing of EA treatment, and the assessment of chronic pruritus behavior.** C** Time course of the chronic scratching behavior in mouse treated with vehicle (control), AD, AD plus EA (AD + EA) or AD plus sham EA (AD + sham EA) (n = 12). **D** Representative images of skin lesions from each group of mice on day8.** E** Time-course of SCORA scores for skin lesions (n = 8). **F** Representative H&E staining photographs from each group. Scale bar = 100 μm. **G** Quantitative assessment of epidermal thickness based on H&E images (n = 8). Data are presented as mean ± S.E.M. Analysis was performed using one-way ANOVA complemented by Tukey's post hoc test for multiple comparisons, *p < 0.05, **p < 0.01, ***p < 0.001

The animal model of AD was developed by daily topical application of 100 μL MC903 to the cervical skin of mice for 7 consecutive days. Subsequently, EA was applied to the acupoints LI11 and LI4 on alternate days, commencing from the second day post-MC903 application. The objective was to assess the therapeutic potential of EA in managing chronic pruritus and mitigating local skin lesions (Fig. 1A, B). In contrast to the control group, the scratching behavior in AD mice increased on the fourth day of MC903 treatment and continued to increase with the time of modeling (Fig. 1C). Notably, EA treatment significantly reduced the scratching behavior on the sixth and eighth days compared to the AD group, while sham EA did not demonstrate a significant impact on this behavior (Fig. 1C).

Consistent with previous studies, AD mice presented obvious clinical symptoms such as scales, erythema, edema, and exfoliation, with elevated SCORAD scores relative to the control (Fig. 1D, E). EA intervention effectively mitigated the skin lesions associated with AD, resulting in reduced SCORAD scores compared to the untreated AD group (Fig. 1D, E). Meanwhile, no significant differences were observed between the AD + Sham EA group and the AD group. HE staining revealed significant epidermal thickening in the AD group, which was relieved by EA treatment (Fig. 1F, G. These data suggested that EA alleviates chronic itch and AD-like symptoms.

### EA inhibited the AD-induced inflammatory cell infiltration and the cytokines signaling pathways

**Fig. 2:**
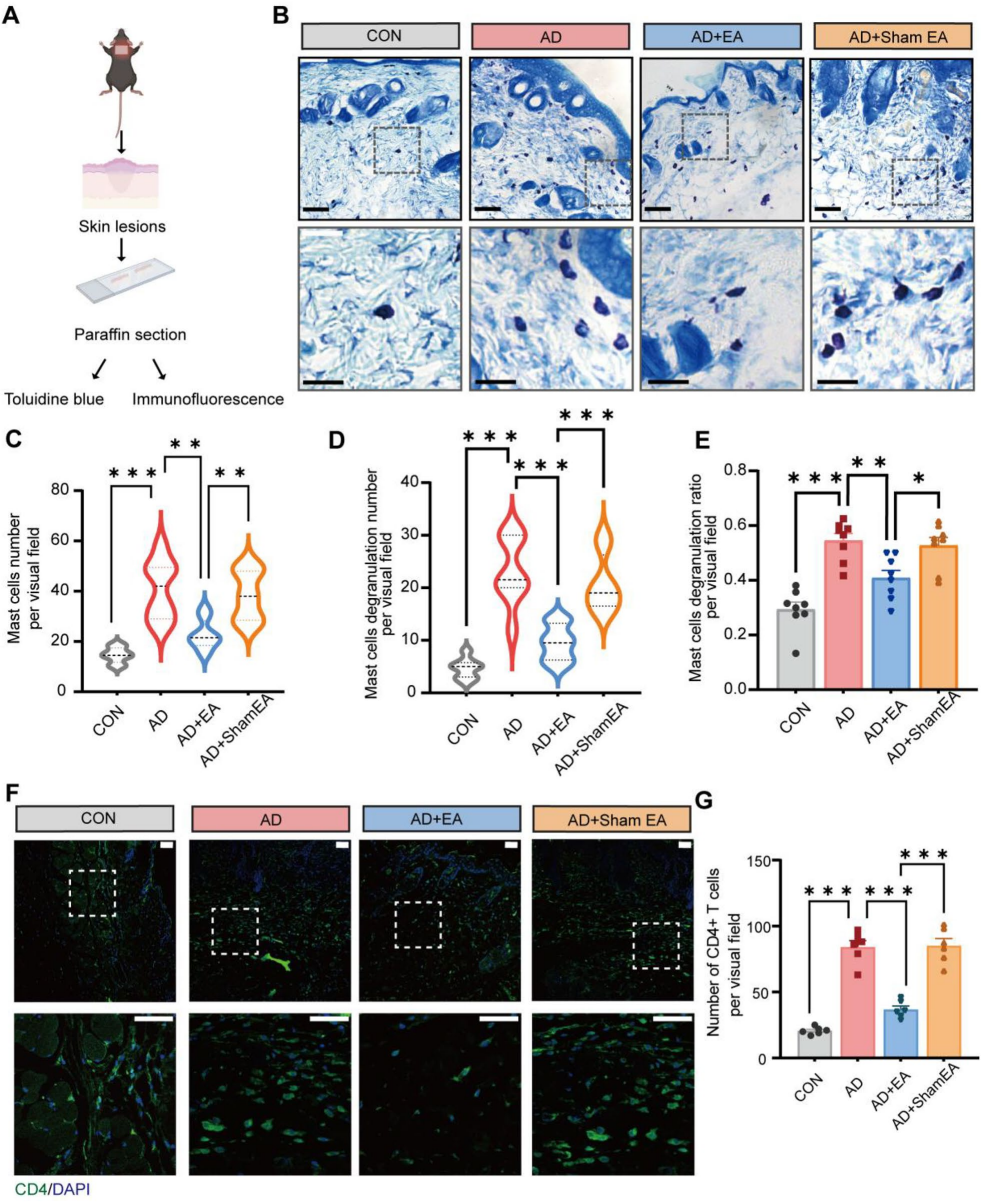
EA inhibited the proliferation of mast cells and CD4^+^ T cells in AD lesional skin.** A** Schematic diagram of toluidine blue and immunofluorescence staining of lesional skin. **B** Representative toluidine blue–stained images of skin lesions. Scale bars (overview) = 100 μm and scale bars (magnified) = 50 μm **C-E** Statistical results of the total number (**C**), the number of degranulation (**D**) and the degranulation rate of mast cells (E) (n = 8). **F** Immunofluorescence images of skin lesions show the CD4^+^ T cells (green). Nuclei of the cells were stained using DAPI (blue). Scale bars (overview) = 100 μm and scale bars (magnified) = 50 μm** G** Aggregate data illustrate the quantity of CD4^+^ T cells within skin sections (n = 6). Data are shown as mean ± S.E.M. Analysis was performed using one-way ANOVA complemented by Tukey's post hoc test for multiple comparisons, *p < 0.05, **p < 0.01, ***p < 0.001

Immune cells are crucial in the development of AD pathogenesis [34], with a particular emphasis on CD4^+^ T cells and mast cells [28]. In this study, we explored the potential therapeutic impact of EA by examining its ability to inhibit the proliferation of inflammatory cells. To identify the specific immune cell populations present in the lesional skin, we respectively performed immunostaining using CD4 markers and toluidine blue to identify CD4^+^ T cells and mast cells (Fig. 2A). The findings showed that AD mice had an elevated total count, degranulation count, and degranulation rate of mast cells compared to the control group (Fig. 2B–E). In contrast, EA treatment suppressed the AD-induced mast cell changes (Fig. 2B–E). Immunofluorescence results revealed an increase in CD4^+^ T cells within the lesional skin of AD mice when contrasted with the controls (Fig. 2F, G. Meanwhile, EA inhibited the increase of CD4^+^ T cells in the lesional skin (Fig. 2F, G).

**Fig. 3:**
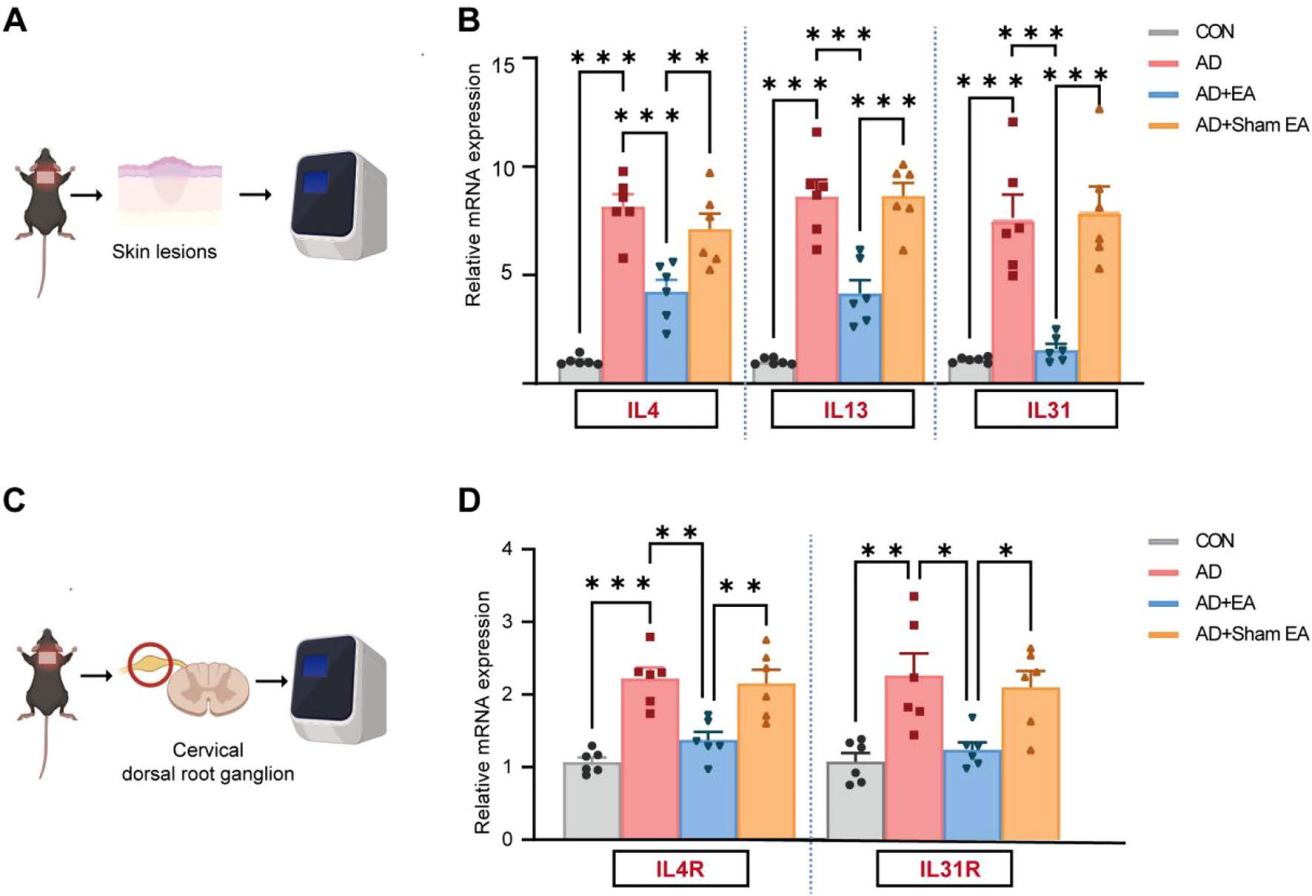
EA inhibited the cytokine release and its receptor activation induced by AD. **A** Schematic diagram of RT-qPCR of skin lesions of mice. **B** Expression levels of IL4, IL13, and IL31 mRNA as determined by RT-qPCR, with all levels normalized to GAPDH. **C** Schematic diagram illustrating RT-qPCR analyses of the cervical segmental DRG. **D** The expression levels of IL4R and IL31R mRNA were assessed using RT-qPCR, normalized to GAPDH. All data are shown as mean ± S.E.M (n = 6 per group). Analysis was performed using one-way ANOVA complemented by Tukey's post hoc test for multiple comparisons, *p < 0.05, **p < 0.01, ***p < 0.001

In the development of AD, cytokines including IL4, IL13, and IL31 play a crucial pro-inflammatory role and contribute to chronic pruritus [20]. Additionally, IL4 receptors (IL4R) and IL31 receptors (IL31R) on the dorsal root ganglion(DRG) are significantly involved in the transmission of itch sensation [35, 36]. Given the established suppressive impact of EA on AD-induced chronic itch, we utilized RT-qPCR to evaluate the expression levels of IL4, IL13, and IL31 in the lesional skin, as well as IL4R and IL31R in the cervical segmental DRG of mice across the experimental groups (Fig. 3A, C). In contrast to the control group, there was an observed increase in the mRNA levels of IL4, IL13 and IL31 in the lesional skin of AD model (Fig. 3B). Compared with AD group, the EA treatment led to a reduction in both the mRNA expression of IL4, IL13 and IL31 in the lesional skin (Fig. 3B). Subsequently, similar analyses were conducted on the DRG. Our findings revealed an upregulation of both IL4R and IL31R mRNA expression in the cervical segmental DRG of AD mice when contrasted to control mice (Fig. 3D). Post-EA intervention, the mRNA levels of IL4R and IL31R in the AD + EA group were observed to be reduced, as opposed to those in AD group (Fig. 3D). Collectively, these findings underscore the capacity of EA therapy to suppress the infiltration of immune cells and AD-related cytokine signaling pathways.

### EA enhanced the expression of CB2R and modulated the endocannabinoid metabolic enzymes within AD skin lesions

**Fig. 4:**
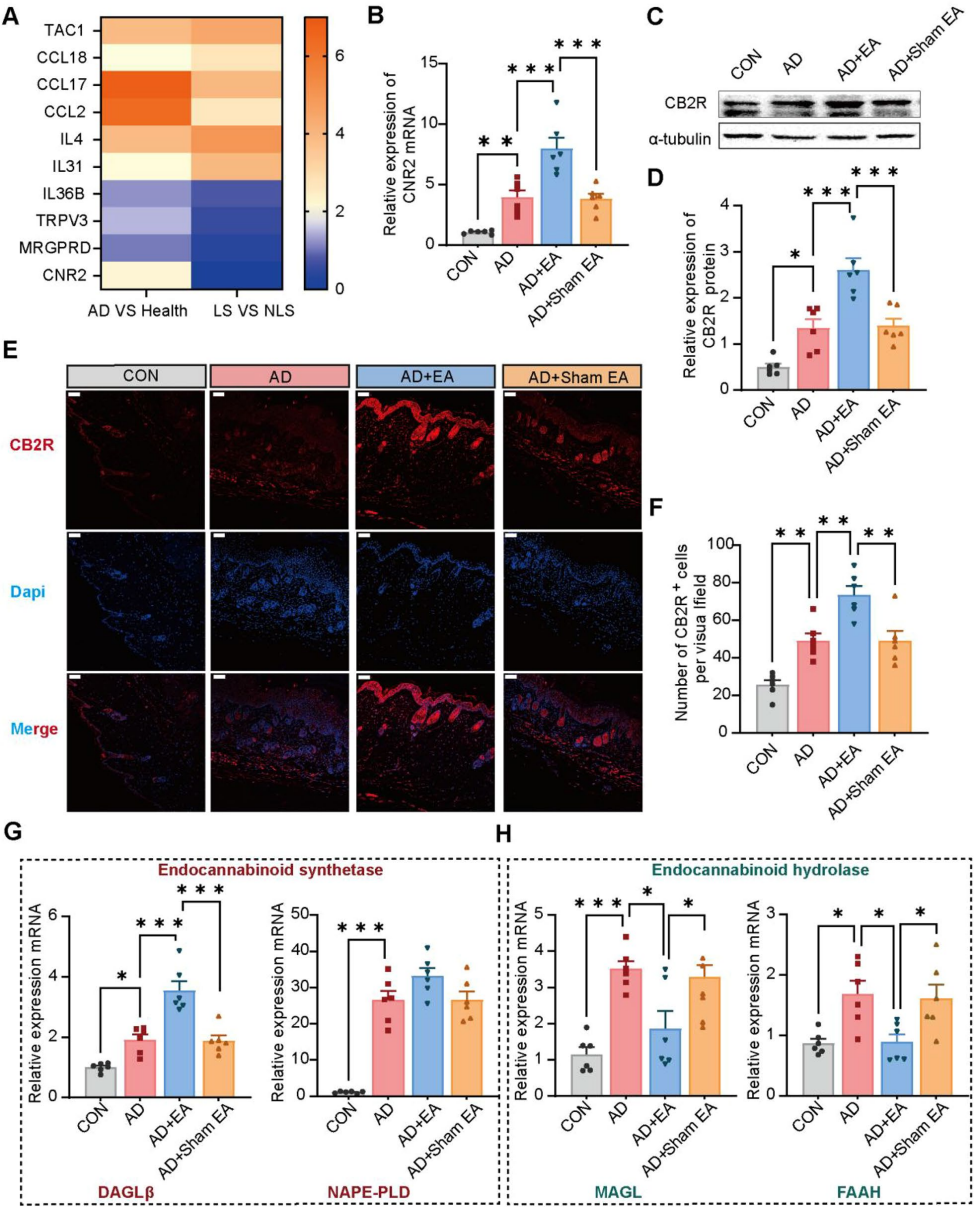
EA further promotes the expression of CB2R and modulates endocannabinoid metabolism in AD skin lesions.** A** Comparative gene expression fold changes (FC) are illustrated on a color scale, with blue indicates underexpression (FC < 2) and yellow indicates overexpression (FC > 2). The comparisons are shown for AD patients’ lesional skin compared to healthy human skin (left panel), and within AD patients between lesional skin (LS) and non-lesional skin (NLS) (right panel).** B** Expression levels of CNR2 mRNA were determined using RT-qPCR, normalization to GAPDH. **C** Representative WB diagram for CB2R. **D** Quantitative analysis of CB2R expression, normalized to α-tubulin.** E** Immunofluorescence images of skin lesions demonstrate the presence of CB2R (red), while nuclei of the cells were stained using DAPI (blue). Scale bars = 100 μm. **F** Summary data illustrate the quantity of CB2R^+^ cells located in the dermis of skin lesions. **G-H** Expression levels of endocannabinoid metabolism-related enzymes, including DAGLβ, NAPE-PLD, MAGL and FAAH mRNA, were assessed via RT-qPCR, normalized to GAPDH. All data are shown as mean ± S.E.M (n = 6 per group). Analysis was performed using one-way ANOVA complemented by Tukey's post hoc test for multiple comparisons, *p < 0.05, **p < 0.01, ***p < 0.001

CB2 receptor (CB2R) agonists are frequently utilized in clinical settings for the management of AD [14]. Additionally, EA has been found to activate CB2R in inflammatory pain sites [18]. To elucidate the potential targets of EA in AD, we conducted a reanalysis of a publicly available AD-related RNA sequencing database cluster [37, 38]. RNA sequencing data revealed an upregulation of CB2R expression in the skin of individuals with AD in comparison to healthy controls (Fig. 4A). Interestingly, a reduction expression of CB2R was noted in lesional skin of AD individuals relative to their own non-lesional skin (Fig. 4A). This observation implies that CB2R might be implicated in the pathophysiology of AD as a regulatory target of EA. To explore this proposition, a range of experiments were conducted to examine the modulation of CB2R expression during EA treatment for AD. To explore this proposition, a range of experiments was conducted to examine the modulation of CB2R expression during EA treatment for AD. The expression of CB2R was assessed by RT-qPCR, WB and immunofluorescence. It indicated that both CB2R mRNA and protein expression were elevated in the AD group relative to the controls (Fig. 4B–F). Furthermore, following EA treatment, a significant upregulation of CB2R mRNA and protein expression was observed compared to the AD group. (Fig. 4B–F).

To explore the potential influence of EA on enhancing CB2R expression, we employed RT-qPCR to analyze the mRNA levels of endocannabinoid synthetase and hydrolase [39]. DAGL β and NAPE-PLD showed increased expression in both the AD and AD + EA groups when contrasted with the control group (Fig. 4G). Additionally, DAGL β expression was found to be further elevated following EA treatment compared to the AD group (Fig. 4G). The endocannabinoid hydrolases MAGL and FAAH were observed to be elevated in both AD and AD + EA groups in comparison to the controls (Fig. 4H). However, after EA intervention, the expression levels of MAGL and FAAH decreased relative to those in the AD group (Fig. 4H). These results indicate that EA therapy might modulate the endocannabinoid system by increasing the expression of CB2R and its associated synthetases while reducing the expression of hydrolase, thereby potentially contributing to its therapeutic effects in AD.

### EA regulates atopic dermatitis-like skin lesions and chronic itching in AD through CB2R

**Fig. 5:**
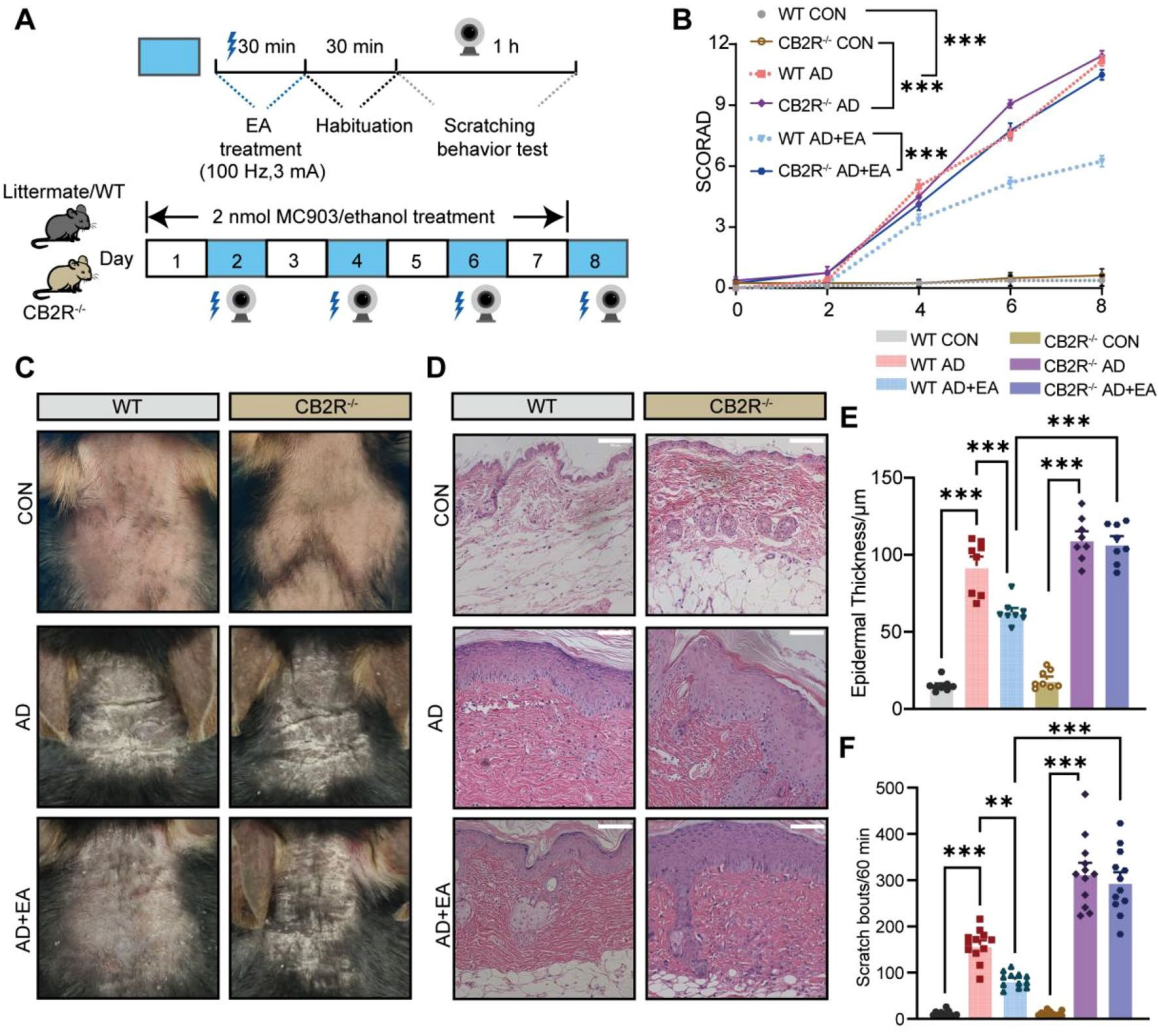
CB2R knockout mitigates the therapeutic efficacy of EA on AD-like lesions and chronic pruritus. **A** The protocol regarding the establishment of the AD model, the EA treatment and the detection time of chronic pruritus behavior in WT and CB2R^−/−^ mice.** B** Time-course of SCORAD scores(n = 8). **C** Representative images of skin lesions from each group of mice on day8. **D** Representative H&E staining images of each group. Scale bar = 100 μm. **E** Quantitative measurement of epidermal thickness based on the H&E images (n = 8). **F** Total number of scratches performed by mice in each group within one hour on day 8 (n = 12). All data are shown as mean ± S.E.M. Analysis was performed using two-way ANOVA complemented by Sidak's test for multiple comparisons, *p < 0.05, **p < 0.01, ***p < 0.001

To determine the contribution of CB2R to the therapeutic efficacy of EA for AD treatment, this study employed CB2R knockout (CB2R^−/−^) mice alongside their wild-type (WT) littermates (Supplementary Fig. 1**)**. Both WT and CB2R^−/−^ mice were independently randomized into three distinct groups: control, AD, and AD + EA. This allocation resulted in a total of six subgroups (WT control, WT AD, WT AD + EA; CB2R^−/−^ control, CB2R^−/−^ AD, CB2R^−/−^ AD + EA). AD + EA mice received EA treatment four times, with a one-day interval between each session, using the same parameters as previously (Fig. 5A). On day 8, both WT and CB2R^−/−^ mice in the AD group exhibited increased SCORAD scores and more severe skin lesions relative to the controls (Fig. 5B, C). After EA intervention, the SCORAD scores of WT AD + EA group decreased and their skin lesions improved, while CB2R^−/−^ AD + EA group did not show the same beneficial effects (Fig. 5B, C). Additionally, CB2R^−/−^ AD + EA group exhibited higher SCORAD scores and more severe skin lesions than WT AD + EA group (Fig. 5B, C). HE staining showed that the AD group of WT and CB2R^−/−^ mice exhibited epidermal thickening compared with the control group (Fig. 5D, F). EA treatment mitigated epidermal thickening in WT mice, whereas no such effect was observed in CB2R^−/−^ mice (Fig. 5D, F). Furthermore, the epidermal thickness of CB2R^−/−^ AD + EA group was significantly greater than that in the WT AD + EA group(Fig. 5D, F). It should be highlighted that CB2R exerts a significant role in the chronic itch behavior elicited by EA treatment of AD. The findings from the pruritus behavioral study reveal that EA treatment significantly diminished the total scratch count within a one-hour period in WT mice, a reduction not observed in CB2R^−/−^ mice (Supplementary Fig. 2, Fig. 5E). These results emphasize the key part that CB2R plays in mediating the antipruritic effect of EA in AD mice.

### CB2 receptor is required for EA to inhibit inflammatory cell infiltration in AD

**Fig. 6:**
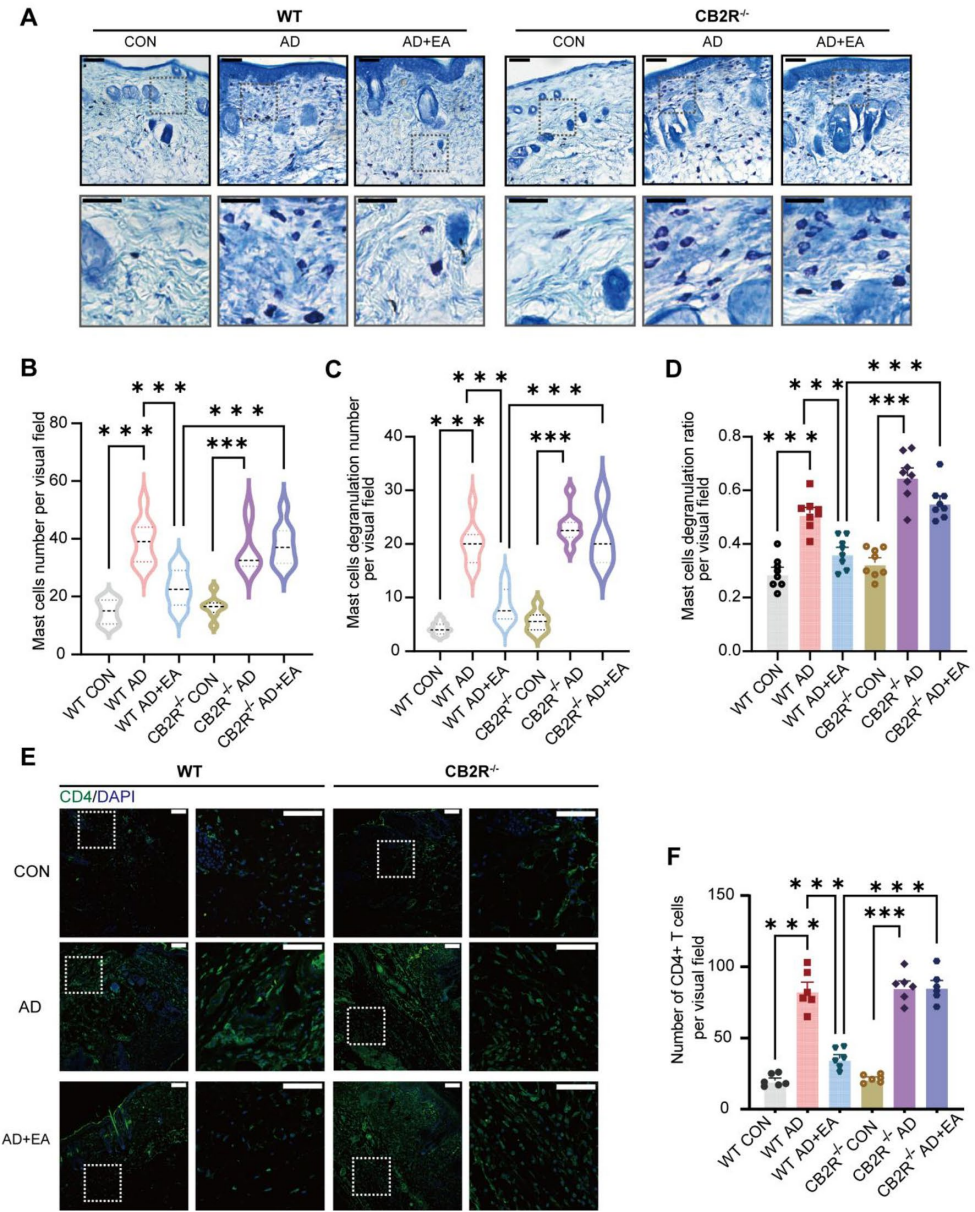
EA influences mast cell and CD4^+^ T cell proliferation in AD through CB2R. **A** Representative toluidine blue–stained images of skin lesions. Scale bars (overview) = 100 μm and scale bars (magnified) = 50 μm. **B-D** Statistical results of the total number (**B**), the number of degranulation (**C**) and the degranulation rate of mast cells (**D**) (n = 8). **E** Immunofluorescence images of skin lesions show the CD4^+^ T cells (green). Nuclei of the cells were stained using DAPI (blue). Scale bars (overview) = 100 μm and scale bars (magnified) = 50 μm **F** Summarized data present the count of CD4^+^ T cells within skin sections (n = 6). All data are shown as mean ± S.E.M. Analysis was performed using two-way ANOVA complemented by Sidak's test for multiple comparisons, *p < 0.05, **p < 0.01, ***p < 0.001

Next, we examined the function of CB2R in EA's therapeutic approach to AD-related inflammation. Toluidine blue results indicated that the induction of AD in WT and CB2R^−/−^ mice led to higher counts of total mast cells, degranulation number, and degranulation rate as compared to the controls (Fig. 6A–D). Following CB2R knockout, EA's efficacy in curbing mast cell proliferation and degranulation in mice with AD was weakened (Fig. 6A–D). Similarly, CD4 immunofluorescence results indicated that AD modeling led to a rise in CD4^+^ T cell counts in both WT and CB2R^−/−^ mice, as compared to the controls. However, following CB2R knockout, the suppressive impact of EA on the CD4^+^ T cell numbers in AD mice was diminished (Fig. 6E, F).

### EA exerts its anti-inflammatory effect by inhibiting ERK phosphorylation via the CB2 receptor

**Fig. 7:**
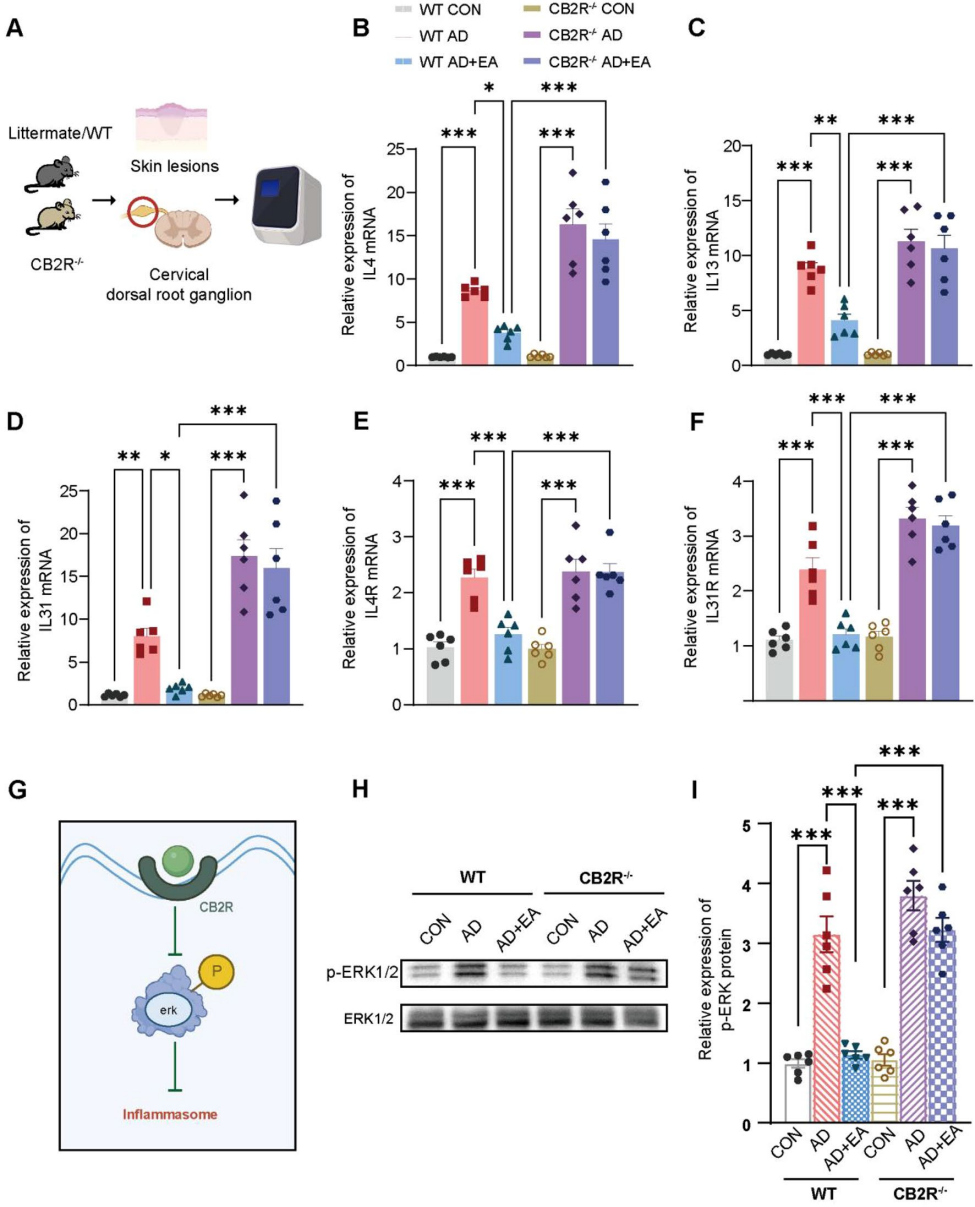
EA modulates cytokine release and receptor activation via CB2R and its downstream ERK pathway in AD mice. **A** Schematic diagram illustrating the procedures for RT-qPCR in each group. **B–F** Expression levels of IL4, IL13, IL31, IL4R and IL31R mRNA as determined by RT-qPCR, normalized to GAPDH. **G** Schematic representation of the role of CB2R activation in modulating inflammatory responses by targeting the ERK phosphorylation. **H** Representative WB results for p-ERK and ERK. **I** Quantitative assessment of p-ERK levels, with expression levels normalized to ERK. All data are shown as mean ± S.E.M (n = 6 per group). Analysis was performed using Two-way ANOVA complemented by Sidak's test for multiple comparisons, *p < 0.05, **p < 0.01, ***p < 0.001

To verify the role of CB2R in EA regulating cytokine signaling pathways in AD mice, lesional skin and cervical DRG samples from WT and CB2R^−/−^ mice were utilized for RT-qPCR analysis (Fig. 7A). RT-qPCR analysis indicated that the absence of CB2R led to the removal of the inhibitory impact of EA on the production of AD-associated cytokines and the activation of receptors (Fig. 7B–F). ERK phosphorylation is known to promote inflammation in AD, while activation of CB2R can inhibit the downstream ERK pathway [40, 41](Fig. 7G). To investigate this, we conducted WB detection of phosphorylated ERK in lesional skin. Our results revealed that in WT mice, EA treatment reduced AD-induced ERK phosphorylation in skin lesions. However, after CB2R knockout, the inhibitory effect of EA on ERK phosphorylation was no longer observed (Fig. 7H, I). These discoveries posit that EA potentially mitigates inflammation in AD mice by modulating the ERK signaling pathway downstream of CB2R, thereby implicating CB2R as a key mediator in the EA’s anti-inflammatory mechanisms.

## Discussion

Clinically, patients with chronic itch frequently experience an uncontrollable itch-scratching cycle, which exacerbates skin damage associated with the condition [42]. Despite this, current therapeutic medications rarely address both itching and inflammation simultaneously. As an alternative therapy, EA is frequently applied in the therapeutic management of AD [43]. Nevertheless, the underlying mechanisms of EA remain unclear, which limits its wider adoption in clinical practice.

In this study, we demonstrate that EA not only alleviates chronic pruritus associated with AD but also significantly inhibits AD-like skin lesions and suppresses the activation of inflammatory pathways. Moreover, EA treatment was found to upregulate the expression of CB2R and endocannabinoid synthetases, while downregulating the expression of endocannabinoid hydrolases. The antipruritic and anti-inflammatory effects of EA were notably attenuated following the genetic ablation of CB2R, and the suppressive impact of EA on ERK phosphorylation was counteracted. These results underscore the essential function of CB2R activation in facilitating the anti-inflammatory and antipruritic properties of EA in the context of AD. Thus, our research offers new perspectives on the therapeutic potential of EA for AD treatment, indicating that EA can alleviate chronic itching by activating CB2R while simultaneously inhibiting inflammatory progression.

AD is a persistent inflammatory condition of the skin, predominantly marked by Th2-mediated immune dysregulation [44]. Mast cells are pivotal in the development of atopic conditions, including AD, where their overproliferation intensifies skin inflammation [26]. Additionally, CD4^+^ T cells are crucial for the progression of AD [28]. In our research, we discovered that EA was capable of suppressing the pathological increase of both mast cells and CD4^+^ T cells. The itch-scratching cycle in AD is further exacerbated by immune cells releasing pro-inflammatory cytokines including IL4, IL13, and IL31 [22]. The binding of these cytokines to their receptors (IL4R, IL31R) in DRG of the cervical segments, thereby exacerbating the symptoms [24]. Furthermore, we observed that EA inhibited cytokine expression in the skin lesions as well as receptor expression in DRG of the corresponding cervical segment. This indicates that EA may disrupt the itch-scratch cycle by modulating cytokines production and receptors expression implicated in this process. It provides a novel insight into the therapeutic potential of EA in managing the intricate interplay of immune mediators in AD.

In this study, we re-analyzed previously published RNA-seq results and found that CB2R was down-regulated in lesional skin as opposed to autogenous non-lesional skin. Interestingly, despite the down-regulation, CB2R expression remained elevated in lesional skin relative to that in healthy controls. This up-regulation in non-lesional skin may signify a promising therapeutic target for AD treatment. Furthermore, while CB2R agonists have been used to treat AD [45], the underlying mechanisms remain unclear. Previous research indicated that EA promotes CB2R activation at the lesion site, thereby exerting an anti-inflammatory effect [18, 46]. Building on this foundation, our recent research findings indicate that in AD mice, EA treatment applied via specific acupoints corresponding to the affected dermatome, initiates a cascade of anti-inflammatory effects. EA stimulates axon reflexes, triggering retrograde neuropeptide release (e.g., CGRP, SP) at peripheral terminals [47]. CGRP can modulate AEA levels in inflammatory microenvironments [48], while SP activates phospholipase C, generating diacylglycerol as a precursor for 2-AG synthesis [49]. These processes are amplified at acupoints, which are densely innervated by Aδ/C fibers, facilitating neuropeptide-mediated crosstalk [50]. Our data confirm that EA upregulates the expression of the endocannabinoid synthetic enzyme DAGLβ while inhibiting the hydrolases MAGL and FAAH. This dual action leads to elevated endocannabinoid levels and enhanced activation of the CB2R. This mechanism aligns with EA’ s ability to modulate the"neuro-metabolic-receptor"axis, offering a multi-target therapeutic strategy for AD.

Notably, CB2R is primarily distributed in peripheral immune cells [51]. In this research, EA’s efficacy on AD-related pruritus was inhibited after the knockdown of CB2R. Additionally, the suppressive effect of EA on cytokine expression in lesional skin and receptors activity in DRG of AD mice was abolished after CB2R knockout. Collectively, these results suggest the essential part that CB2R plays in facilitating the antipruritic and anti-inflammatory effects of EA in the context of AD.

The ERK pathway, as a downstream component of the CB2R, is integral to the control of cell division and is triggered by mitogens and growth factors [52]. This pathway is essential for controlling cell proliferation and Th2 cell differentiation [53]. Previous research has shown that AD is associated with increased ERK phosphorylation in lesional skin [40]. Consistent with these findings, EA has been shown to inhibit ERK phosphorylation in skin, potentially contributing to its therapeutic impact on AD. Notably, the inhibition of ERK phosphorylation by EA was reversed following the knockout of CB2R.

## Conclusion

**Fig. 8:**
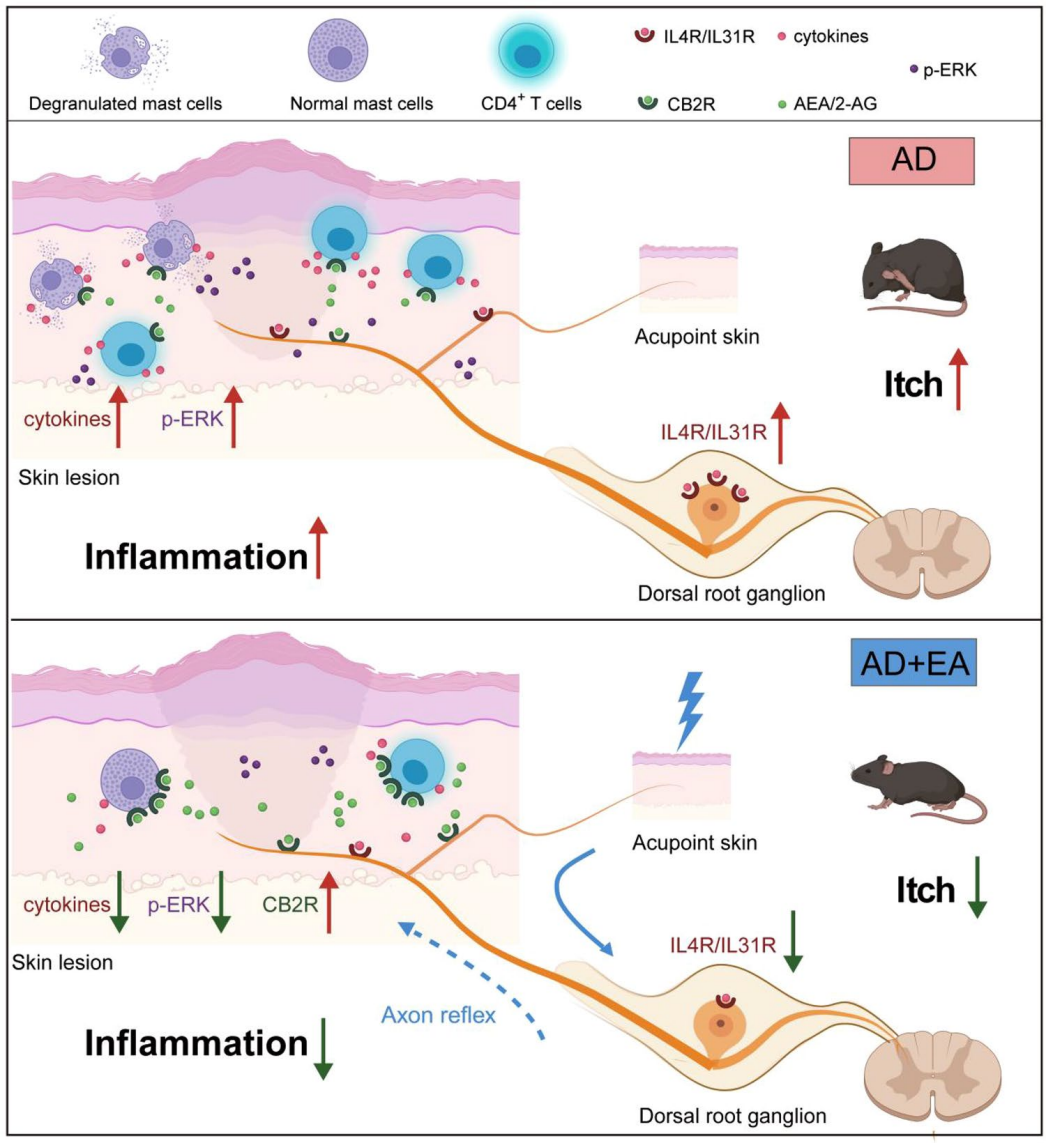
Schematic diagram of this study's findings on the impact of EA on AD Mice. This schematic diagram presents the pathophysiological alterations in AD and the therapeutic interventions of EA treatment. Initially, upon the induction of AD, there is an escalation in the number of mast cells and CD4^+^ T cells within the skin lesions, along with upregulation of cytokines IL4, IL13, and IL31, and their receptors IL4R and IL31R in the cervical DRG. These changes are linked to the exacerbation of chronic itching and skin inflammation. Despite an upregulation of the CB2R, the endogenous levels are not adequate to effectively inhibit ERK phosphorylation, a key driver of inflammatory processes (left side). In contrast, EA treatment, applied via specific acupoints corresponding to the affected dermatome, initiates a cascade of anti-inflammatory effects. EA stimulates axon reflexes that enhance the synthesis of endocannabinoids and reduce their degradation in the lesional skin tissue. This results in elevated levels of the primary endocannabinoids such as AEA and 2-AG, which in turn upregulate the expression of CB2R. Stimulation of CB2R by endocannabinoids leads to a significant reduction in ERK hyperphosphorylation, an essential process in curbing inflammatory response. As a result, EA treatment diminishes the number of mast cells and CD4^+^ T cells in lesional skin, reduces the expression of pro-inflammatory cytokines IL4, IL13, and IL31, and decreases the expression of IL4R and IL31R in the cervical DRG. Collectively, these effects contribute to the alleviation of itching and the inhibition of inflammation development (right side)

These novel insights significantly enhance our comprehension of how EA achieves its therapeutic effects in the context of AD. EA exerts therapeutic effects on persistent itch and skin inflammation in AD mice by activating CB2R, thereby inhibiting mast cell and CD4 + T cell proliferation and the expression of associated inflammatory factors, as well as downstream ERK phosphorylation (Fig. 8). The detailed elucidation of these pathways not only substantiates the clinical application of EA for symptom management in AD but also establishes a solid scientific groundwork for the broader incorporation of EA into standard medical practice.

## Supplementary Information

Supplementary material 1.

## Data Availability

All the data supporting the findings of this study are available within the article and from the corresponding author upon reasonable request.

## Abbreviations

AD: Atopic dermatitis
EA: Electroacupuncture
CB2R: CB2 receptor
NAPE-PLD: N-acyl phosphatidylethanolamine-phospholipase D
DAGL β: Diacylglycerol lipase β
FAAH: Fatty acid amide hydrolase
MAGL: Monoacylglycerol lipase
ERK: Extracellular signal-regulated kinase
CB2R^−^^/^^−^: CB2R knockout
SCORAD: Scoring Atopic Dermatitis
DRG: Dorsal root ganglion
H&E: Hematoxylin and eosin
