# Supplementary material for: Electroacupuncture exerts antipruritic and anti-inflammatory effects on atopic dermatitis by activating CB2 receptor
Source: Chin Med. 2025 May 26;20:72. doi: 10.1186/s13020-025-01102-4 (PMC12105266; doi:10.1186/s13020-025-01102-4)
Supplement: Supplementary file 1 — Supplementary material 1. [file 13020_2025_1102_MOESM1_ESM.pdf]

Supplementary Table S1. Primer Sequences for PCR Genes

Gene name	Forward 5’ –3’	Reverse 5’–3’
IL4	TACCAGGAGCCATATCCACGGATG	TGTGGTGTTCTTCGTTGCTGTGAG
IL13	CTTGCTTGCCTTGGTGGTCTC	GGAGTCTGGTCTTGTGTGATGTTG
IL31	GCCTACCCTGGTGCTGCTTTG	TGTGCTATGATGACCGAGATGTTGG
IL4R	CTGTGGCTGCTGCTACGATGAC	GAGGTTGGCTTCTGGTGGTATTCC
IL31R	AGGAAGGTGCGATTGTTGTGGAAG	TTCATACTGCTGGGTGGTGATGTTG
CNR2	CGTGATCTTCGCCTGCAAC	GTCAACAGCGGTTAGCAGCA
DAGLβ	ACCGTCATTGTCAGCTGGAT	GCTGTTACTGTCCAGGTGCT
NAPE-PLD	GAAGAGCCACCGCGTTTTTAG	CCTCGGCGAGAAGTTGTATTC
MAGL	CCTCACCTGGTCAATGCAGA	ACAAAGATGAGGGCCTTGGG
FAAH	GGCAATGAACTTGGACGTGG	TGTAGCTGATAGCCCCTGTG

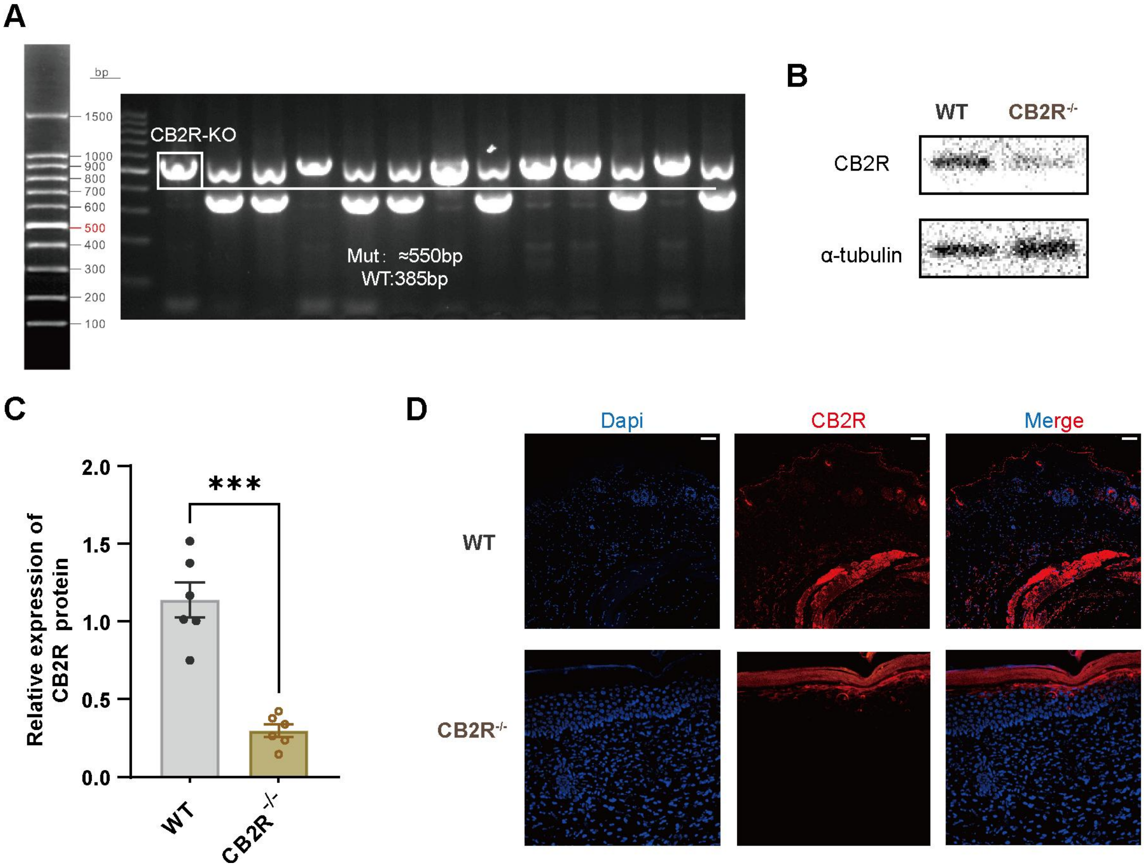

Supplementary Figure S1. Characterization of CB2R expression in the cervical skin of CB2R$^{-/-}$ Mice. (A) The agarose gel map illustrates the PCR-amplified DNA fragments from CB2R$^{-/-}$ mice. (B) Representative WB diagram for CB2R. (C) Quantitative analysis of CB2R expression, with expression levels normalized to α-tubulin. (D) Immunofluorescence images of skin lesions demonstrate the presence of CB2R (red), while nuclei of the cells were stained using DAPI (blue). Scale bars=100 µm. Data are shown as mean ± S.E.M (n = 6 per group). *** $p < 0.001$, as determined by an unpaired *t*-test.

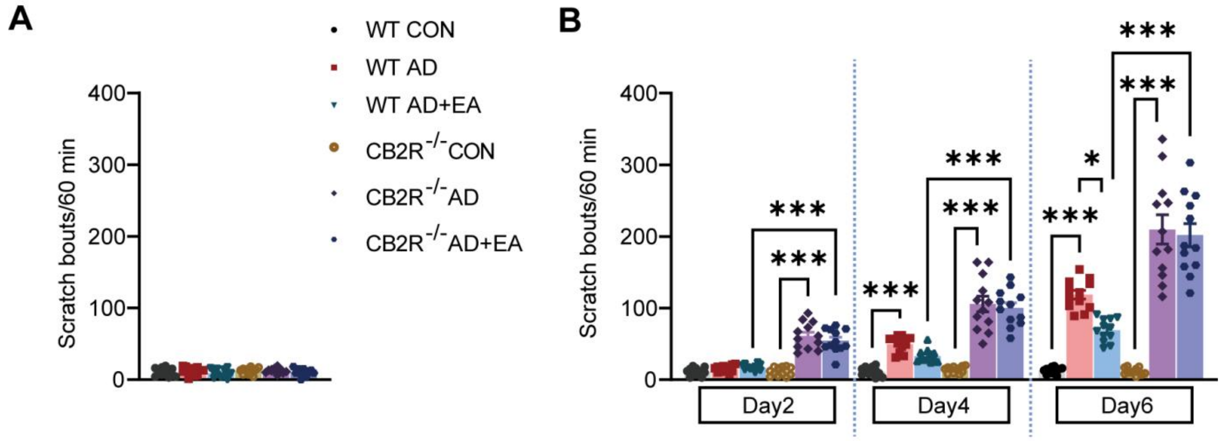

Supplementary Figure S2. The Scratching behavior in CB2R$^{-/-}$ and WT mice. (A) The baseline scratch bouts of CB2R$^{-/-}$ and WT mice. (B) Time course of the chronic scratching behavior of in CB2R$^{-/-}$ and WT mice. Data are shown as mean ± S.E.M (n = 12 per group). Analysis was performed using two-way ANOVA complemented by Sidak's test for multiple comparisons, *$p < 0.05$, **$p < 0.01$, ***$p < 0.001$.
